# The Invisible Struggle: The Psychosocial Aspects of Polycystic Ovary Syndrome

**DOI:** 10.7759/cureus.51321

**Published:** 2023-12-30

**Authors:** Deepika Dewani, Pravin Karwade, Kalyani S Mahajan

**Affiliations:** 1 Department of Obstetrics and Gynecology, Jawaharlal Nehru Medical College, Datta Meghe Institute of Higher Education and Research, Wardha, IND; 2 Department of Obstetrics and Gynecology, Symbiosis Medical College for Women, Symbiosis International University, Pune, IND

**Keywords:** advocacy, holistic care, mental health impacts, stigma, psychosocial aspects, pcos (polycystic ovary syndrome)

## Abstract

Polycystic ovary syndrome (PCOS) is a prevalent endocrine disorder with multifaceted manifestations, affecting both physiological and psychosocial aspects of affected individuals. This abstract provides a succinct overview of the hormonal underpinnings in the pathogenesis of PCOS, focusing on altered luteinizing hormone (LH) action, insulin resistance, and hyperandrogenism. A prevailing theory suggests that insulin resistance exacerbates hyperandrogenism by influencing the synthesis of sex hormone-binding globulin and increasing androgen production from adrenal and ovarian sources. PCOS diagnosis relies on specific criteria related to hyperandrogenism, ovulatory dysfunction, and the presence of polycystic ovaries. Beyond its physical symptoms, PCOS profoundly impacts women's mental health and quality of life. The prevalence of PCOS underscores the urgency of understanding its hormonal intricacies. Insulin resistance and hyperandrogenism, particularly in the context of sex hormone-binding globulin suppression, play a central role in PCOS pathogenesis. Recognizing the key role of hormones, particularly insulin resistance and hyperandrogenism, provides a foundation for targeted interventions and treatment strategies. A comprehensive approach to PCOS must consider both its physiological and psychosocial dimensions to address the challenges faced by affected individuals.

## Introduction and background

Polycystic ovary syndrome (PCOS) stands as one of the most prevalent endocrine disorders affecting individuals of reproductive age. Characterized by hormonal imbalances, irregular menstruation, and the presence of cysts on the ovaries, PCOS extends beyond its physiological manifestations, permeating into the psychosocial realm of those affected [1]. PCOS is a multifaceted health condition that transcends the confines of gynecological concerns. As an endocrine disorder, it manifests through disruptions in hormonal levels, most notably androgen excess, insulin resistance, and irregular ovulation. Clinically, the syndrome is diagnosed based on the Rotterdam criteria, which encompass hyperandrogenism, irregular menstrual cycles, and the presence of polycystic ovaries on ultrasound. The physical consequences of PCOS have been extensively documented, yet the effects on mental health and social well-being are equally profound [2].

While the physical symptoms of PCOS are often the primary focus of clinical attention, the psychosocial aspects of this syndrome play a pivotal role in the overall well-being of affected individuals. The invisible struggle that accompanies PCOS involves coping with societal misconceptions, facing stigmatization, and navigating the often complex interplay between physical and mental health. Recognizing and addressing these psychosocial dimensions is imperative for comprehensive, patient-centered care [3].

The purpose of this review is to delve into the psychosocial intricacies of PCOS, shedding light on the often-overlooked aspects that significantly impact the lives of those affected. By synthesizing existing literature, exploring the link between PCOS and mental health, and investigating the role of societal perceptions, this review aims to contribute to a deeper understanding of the holistic implications of PCOS. Furthermore, we highlight the importance of incorporating psychosocial considerations into clinical practice, advocating for a more comprehensive and compassionate approach to PCOS care.

## Review

Understanding PCOS

PCOS is a complex condition diagnosed by the presence of at least two of the following three criteria: hyperandrogenism, ovulatory dysfunction, and polycystic ovaries [1,4,5]. The condition is the most common endocrinopathy among reproductive-aged women in the United States, affecting approximately 7% of female patients [5]. The exact etiology of PCOS is thought to emerge from a complex interaction of genetic and environmental traits, with evidence indicating a strong correlation between familial factors and the presence of PCOS [5].

Prevalence and Demographics

PCOS affects women's mental health and quality of life, with anxiety and depression being prevalent issues among patients [6]. Studies have shown that women with PCOS tend to experience mildly elevated anxiety and depression [6]. The condition is stigmatizing and affects a woman's identity, mental health, and quality of life [6]. The prevalence of anxiety and depressive disorders among women with PCOS ranges from 28% to 39% for anxiety and 11% to 25% for depression [6].

Physical Symptoms and Health Implications

The clinical presentation of PCOS is characterized by a diverse array of symptoms, contributing to its variable nature among affected individuals. One hallmark of PCOS is the presence of signs indicating hyperandrogenism, a condition marked by elevated levels of male hormones, particularly androgens. The diagnostic criteria for PCOS often involve the observation of hyperandrogenism along with additional factors, such as irregular menstrual cycles, the presence of ovarian cysts, and related reproductive concerns [5].

The physical manifestations of PCOS are wide-ranging and can be observed in various ways. Hirsutism, characterized by excessive hair growth, is a common manifestation resulting from elevated androgen levels. Another notable physical symptom is insulin resistance, a condition where cells exhibit reduced responsiveness to insulin, leading to metabolic disturbances and increased risk of other health issues, such as type 2 diabetes. Individuals with PCOS often experience acne, a skin condition. Additionally, irregular menstrual periods, including oligomenorrhea (infrequent menstrual periods) or amenorrhea (absence of menstrual periods), are frequently observed [4]. The impact of PCOS extends beyond its immediate physical symptoms, influencing long-term health. Individuals with PCOS face an increased risk of developing type 2 diabetes due to insulin resistance. Additionally, the syndrome is associated with a higher likelihood of experiencing high blood pressure. Importantly, PCOS poses a potential risk factor for endometrial cancer, underscoring the need for vigilant monitoring and comprehensive healthcare for individuals with this syndrome [4].

Role of Hormones in PCOS

The role of hormones in PCOS is pivotal in understanding its pathogenesis. Altered luteinizing hormone (LH) action, insulin resistance, and a potential predisposition to hyperandrogenism have been identified as key components in the development of PCOS [5]. One proposed theory suggests that underlying insulin resistance plays a crucial role in exacerbating hyperandrogenism. This occurs through the suppression of sex hormone-binding globulin synthesis and an increase in adrenal and ovarian synthesis of androgens, ultimately leading to elevated androgen levels [5]. The diagnostic criteria for PCOS are intricately linked to these hormonal imbalances, encompassing factors related to hyperandrogenism, ovulatory dysfunction, and the presence of polycystic ovaries. The condition not only manifests with physical symptoms but also significantly impacts women's mental health and overall quality of life. The prevalence of PCOS is notable, further underscoring the importance of understanding its hormonal underpinnings [5]. The interplay between these hormonal factors not only contributes to the characteristic symptoms that define PCOS but also poses long-term health implications. Recognizing the central role of hormones, especially insulin resistance and hyperandrogenism, provides a foundation for developing targeted interventions and treatment strategies aimed at addressing the hormonal imbalances that underlie the complex landscape of PCOS [5]. Table 1 describes the role of hormones in PCOS. 

**Table 1:** Role of hormones in polycystic ovary syndrome (PCOS)

Hormone	Role in PCOS	Mechanism/relation
LH (luteinizing hormone)	Altered LH action is associated with PCOS pathogenesis.	Dysregulation of LH contributes to hormonal imbalances in PCOS.
Insulin	Insulin resistance is a key factor in PCOS development.	Underlying insulin resistance exacerbates hyperandrogenism and affects androgen synthesis.
Androgens	Hyperandrogenism is a characteristic feature of PCOS.	Increased adrenal and ovarian synthesis of androgens, influenced by insulin resistance.
SHBG (sex hormone–binding globulin)	Suppression of SHBG synthesis is linked to PCOS.	Insulin resistance suppresses SHBG synthesis, contributing to elevated androgen levels.

The invisible struggle: the stigma surrounding PCOS

Social Misconceptions and Stereotypes

The stigmatization of PCOS often begins with prevalent social misconceptions and stereotypes that perpetuate a narrow understanding of this complex syndrome. Misguided beliefs surrounding PCOS contribute to the perpetuation of harmful stereotypes, such as the notion that it is solely a reproductive issue or that it is directly linked to lifestyle choices. These misconceptions not only hinder public awareness but also fuel the marginalization of individuals grappling with PCOS, making it essential to dismantle these unfounded beliefs for a more informed and empathetic societal perspective [7].

Impact of Cultural and Societal Norms

Cultural and societal norms further compound the invisible struggle experienced by those with PCOS. Different cultures may hold distinct views on fertility, body image, and gender roles, thereby influencing how PCOS is perceived within various communities. The pressure to conform to societal expectations regarding femininity and fertility can exacerbate the emotional toll on individuals with PCOS, leading to feelings of inadequacy and isolation. Understanding and addressing these cultural influences are crucial for fostering an environment that supports individuals affected by PCOS [8].

Challenges in Diagnosis and Recognition

The journey to a PCOS diagnosis is often riddled with challenges, contributing to the invisible nature of the struggle. Healthcare providers may encounter difficulties recognizing the syndrome due to its heterogeneous presentation and the absence of a definitive diagnostic test. This diagnostic uncertainty can lead to delayed identification and intervention, leaving individuals to grapple with symptoms and uncertainties. Addressing these challenges in the diagnostic process is pivotal for ensuring timely and accurate support for those navigating the complexities of PCOS [9].

The Need to Dispel Myths and Misinformation

A cornerstone in combating the stigma surrounding PCOS lies in the urgent need to dispel prevailing myths and misinformation. Myths perpetuated by outdated sources, social media, and even healthcare misconceptions contribute to a climate of misunderstanding. Common misconceptions, such as PCOS being solely a reproductive issue or a consequence of lifestyle choices, need to be debunked through accurate and accessible information. Empowering individuals with knowledge is crucial to dispelling myths and instrumental in fostering a sense of agency and control over their health [10].

Mental health impacts of PCOS

Depression and Anxiety

The intersection of PCOS and mental health is complex, with individuals often grappling with heightened risks of depression and anxiety. The hormonal imbalances characteristic of PCOS can contribute to mood disturbances, amplifying the risk of depressive symptoms and anxiety disorders. Beyond the physiological factors, the emotional toll of managing a chronic condition, coupled with societal pressures and potential fertility concerns, can further contribute to the heightened prevalence of depression and anxiety among those with PCOS. Recognizing and addressing these mental health implications is crucial for fostering a holistic approach to PCOS care [11]. PCOS is associated with an increased risk of a diagnosis of depression, anxiety, bipolar disorder, and obsessive-compulsive disorder [12]. Studies have shown that women with PCOS are more likely to have a clinical diagnosis of depression and experience worse symptoms of depression and anxiety [12]. The prevalence of anxiety and depressive disorders among women with PCOS ranges from 28% to 39% for anxiety and 11% to 25% for depression [6].

Body Image Issues and Self-Esteem

PCOS has profound effects on body image and self-esteem, as individuals may contend with physical symptoms such as weight gain, acne, and hirsutism. Societal beauty standards often exacerbate these challenges, perpetuating unrealistic expectations that can be particularly distressing for individuals with PCOS. The visible manifestations of the syndrome can lead to a negative self-perception and, in some cases, contribute to the development of eating disorders. A comprehensive understanding of the impact of PCOS on body image is essential for implementing supportive interventions that address both the physical and psychological aspects of well-being [13].

Relationship and Fertility Concerns

The psychosocial impact of PCOS extends to interpersonal relationships, with potential ramifications on both romantic and familial connections. Fertility concerns, a common worry for individuals with PCOS, can strain relationships and contribute to emotional distress. The challenges associated with fertility treatments, coupled with the uncertainty surrounding conception, can further intensify the strain on relationships. Acknowledging and addressing the emotional toll on both individuals and their partners is crucial for providing holistic support and fostering open communication within relationships affected by PCOS [14].

The Link Between Hormonal Fluctuations and Mood

Hormonal fluctuations in PCOS, particularly irregular menstrual cycles, have been identified as strong predictors of mental health issues, including anxiety and depression [15]. The condition's symptoms, such as body hair and menstrual problems, have been found to strongly predict anxiety, while obesity is associated with hostility [15]. PCOS has significant mental health implications, including an increased risk of depression, anxiety, and other psychiatric disorders, as well as challenges related to body image, self-esteem, relationships, and fertility. Understanding and addressing these impacts are crucial for providing comprehensive care to individuals with PCOS.

Unraveling stigma: strategies for awareness and education

Public Awareness Campaigns

Public awareness campaigns play a pivotal role in dismantling the stigma surrounding PCOS by disseminating accurate information and fostering understanding. These campaigns can utilize various media platforms, including social media, television, and community events, to reach a diverse audience. The focus should be on debunking myths, highlighting the multifaceted nature of PCOS, and promoting inclusivity. By engaging the public in meaningful conversations, these campaigns contribute to a more informed and empathetic society, ultimately reducing the stigma associated with PCOS [16].

Education in Healthcare Settings

To address stigma effectively, it is essential to equip healthcare professionals with the knowledge and skills necessary to recognize and respond to the psychosocial aspects of PCOS. Integrating education on the mental health implications of PCOS into medical training programs ensures that healthcare providers are well-prepared to offer holistic care. This includes fostering an understanding of the challenges individuals with PCOS face, the impact on mental health, and the importance of cultivating a supportive and empathetic healthcare environment [17].

Advocacy for Accurate Media Representation

Media representation significantly influences societal perceptions. Advocating for accurate and nuanced portrayals of PCOS in the media is crucial for challenging stereotypes and dispelling misinformation. Collaborating with media outlets, influencers, and content creators to ensure that narratives around PCOS are inclusive, respectful, and reflective of the diverse experiences of those affected can contribute to reducing stigma. By portraying PCOS as a complex health condition rather than a mere cosmetic concern, the media can play a pivotal role in reshaping public perceptions [18].

Empowering Individuals Through Knowledge

Empowering individuals with PCOS through knowledge is a critical component in dismantling stigma. Providing accessible and accurate information about the syndrome helps individuals understand their condition, manage expectations, and advocate for their well-being. This can be achieved through educational resources, support groups, and online platforms that facilitate the exchange of information and experiences. Empowered individuals are better equipped to challenge stigmatizing attitudes, advocate for themselves within healthcare settings, and contribute to broader awareness efforts [19].

Support strategies for individuals with PCOS

Healthcare Provider Collaboration

Collaboration with healthcare providers is paramount in providing comprehensive support for individuals with PCOS. Establishing a collaborative approach involves fostering open communication between patients and healthcare professionals, including gynecologists, endocrinologists, nutritionists, and mental health practitioners. By addressing both the physiological and psychosocial aspects of PCOS, healthcare providers can tailor treatment plans to individual needs, offering a holistic approach that considers the diverse challenges associated with the syndrome [20].

Peer Support Groups

Peer support groups offer a valuable platform for individuals with PCOS to connect, share experiences, and exchange coping strategies. These groups create a sense of community, reducing feelings of isolation and providing emotional support. Peer support can be particularly impactful in addressing psychosocial aspects, as individuals can relate to one another's challenges and successes. Facilitating the formation of local or online peer support groups ensures that individuals with PCOS have a supportive network to turn to throughout their journey [21].

Mental Health Interventions

Incorporating mental health interventions as part of PCOS care is essential for addressing the emotional toll of the syndrome. Psychosocial support, counseling, and therapy can assist individuals in navigating the complex emotions associated with PCOS, including anxiety, depression, and body image concerns. Mental health professionals can collaborate with healthcare providers to develop tailored interventions that recognize the unique mental health needs of individuals with PCOS, contributing to a more holistic and patient-centered approach to care [22].

Lifestyle Modifications and Holistic Approaches

Lifestyle modifications, including dietary changes, regular exercise, and stress management, can be crucial in managing PCOS symptoms and improving overall well-being. Nutritionists and fitness experts can collaborate with healthcare providers to develop personalized plans that address PCOS's physical and mental health aspects. Additionally, holistic approaches such as mindfulness, yoga, and acupuncture may complement traditional medical interventions, offering individuals with PCOS a well-rounded toolkit for managing their health [23].

Integrating psychosocial support into PCOS care

Multidisciplinary Approach in Healthcare

Adopting a multidisciplinary approach within healthcare settings is essential for effectively integrating psychosocial support into PCOS care. This involves collaboration among healthcare professionals, including gynecologists, endocrinologists, mental health practitioners, and nutritionists. By fostering communication and teamwork, a multidisciplinary approach ensures that individuals with PCOS receive holistic care that addresses their health's physical and psychosocial dimensions. This collaborative model acknowledges the interconnected nature of PCOS and emphasizes a comprehensive treatment plan [24].

Counseling and Mental Health Services

Incorporating counseling and mental health services as routine components of PCOS care is critical for addressing the emotional impact of the syndrome. Mental health professionals, such as psychologists or counselors, can provide support in managing stress, anxiety, and depression associated with PCOS. Integrating regular mental health check-ins into the care protocol allows for proactive identification of psychosocial challenges and facilitates timely interventions. By making mental health services readily accessible, healthcare providers contribute to a more patient-centered and comprehensive approach to PCOS care [25].

Patient-Centered Care Models

Patient-centered care models prioritize individuals' unique needs, preferences, and experiences. Tailoring PCOS care to be patient-centered involves actively involving individuals in decision-making, setting realistic treatment goals, and considering their psychosocial well-being. This approach fosters a collaborative relationship between healthcare providers and patients, empowering individuals to participate actively in their care journey. Patient-centered care models recognize the importance of addressing not only the physical symptoms of PCOS but also the emotional and social aspects that significantly impact overall well-being [26].

Importance of Personalized Treatment Plans

Recognizing the heterogeneity of PCOS presentations, personalized treatment plans are indispensable in providing adequate psychosocial support. Tailoring interventions to each individual's specific needs, concerns, and goals acknowledges the variability in symptom severity and psychosocial impact. Personalized treatment plans may include a combination of medical interventions, mental health support, lifestyle modifications, and educational resources. This individualized approach ensures that individuals with PCOS receive care that resonates with their unique experiences and fosters a sense of agency in managing their health [27].

Future directions and research needs

Gaps in Current Understanding

Despite significant progress in our understanding of PCOS, there still needs to be more in our comprehension of both its physiological and psychosocial dimensions. Future research should delve into the intricacies of PCOS, exploring the underlying mechanisms of hormonal dysregulation, the interplay between genetic and environmental factors, and the long-term health consequences. Additionally, there is a need for more comprehensive studies that examine the psychosocial impact of PCOS, including the experiences of diverse populations, the influence of cultural factors, and the effectiveness of current interventions. Identifying and addressing these knowledge gaps is crucial for advancing our understanding of PCOS and refining treatment approaches [28].

Emerging Research on Psychosocial Impacts

As the awareness of the psychosocial impacts of PCOS grows, there is a need for continued research to explore emerging areas of interest. Investigating the relationship between PCOS and mental health, body image, and quality of life can provide valuable insights into the lived experiences of affected individuals. Furthermore, understanding the psychosocial implications at different life stages, such as adolescence, pregnancy, and menopause, can contribute to more targeted interventions. Emerging research should also explore the role of technology, such as mobile applications and virtual support platforms, in enhancing psychosocial support for individuals with PCOS [29].

Advocacy for Increased Funding and Attention

Advocacy for increased funding and attention is essential to propel PCOS research and awareness initiatives forward. Securing resources for large-scale, longitudinal studies can provide a more comprehensive understanding of PCOS, including its long-term impacts and potential preventive measures. By advocating for increased funding, researchers can explore novel approaches to psychosocial support and intervention strategies and develop more patient-centric care models. Additionally, raising awareness among policymakers, healthcare providers, and the general public is crucial for prioritizing PCOS on the public health agenda and reducing the stigma associated with this syndrome [30].

## Conclusions

In conclusion, our exploration of PCOS has illuminated the intricate interplay between its physiological complexities and the often-overlooked psychosocial dimensions. From the pervasive stigma surrounding PCOS to the profound impacts on mental health, it is evident that a comprehensive approach is imperative for the holistic care of individuals affected by this syndrome. Throughout this review, we emphasized the need for a paradigm shift, urging healthcare providers, policymakers, and researchers to recognize and address the invisible struggles individuals with PCOS endure. It is not enough to focus solely on medical interventions; a comprehensive model must embrace psychosocial support as an integral element of care. This call to action implores the healthcare community to advocate for inclusive, patient-centered care models that prioritize the diverse needs of those with PCOS. By dispelling stigma, acknowledging the gaps in our understanding, and emphasizing the importance of psychosocial aspects, we can forge a path toward a future where individuals with PCOS receive compassionate, holistic care that empowers them to navigate the challenges of this complex syndrome with resilience and confidence.
